# Interdisciplinary medical education practices: building a case-driven interdisciplinary simulation system based on public datasets

**DOI:** 10.1186/s12909-025-07631-8

**Published:** 2025-07-11

**Authors:** Kangli Qiu, Tianshu Zeng, Wenfang Xia, Miaomiao Peng, Wen Kong

**Affiliations:** 1 Department of Endocrinology, Union Hospital, Tongji Medical College, Huazhong University of Science and Technology, Wuhan, Hubei China; 2 Diabetes and Metabolic Disease Clinical Research Center of Hubei Province, Wuhan, China; 3 Hubei Key Laboratory of Metabolic Abnormalities and Vascular Aging, Wuhan, China; 4 Hubei Branch of National Center for Clinical Medical Research of Metabolic Diseases, Wuhan, China

**Keywords:** Practice teaching, Dataset, Case-driven, Simulation, Interdisciplinary, Medicine + X

## Abstract

**Background:**

Recent advancements in medical education underscore the importance of training professionals who are proficient in multiple disciplines. This study aims to develop clinical data analysis cases centered around diseases by utilizing public datasets, and to investigate the establishment of a “medicine + X” simulation practice system within the framework of interdisciplinary disciplines.

**Methods:**

From a multi-disciplinary perspective, we designed a cross-disciplinary “medicine + X” subject simulation practice system based on three dimensions: data, case, and simulation. This system comprises three parts: dataset classification, dataset modeling, and dataset clinical analysis. The entire interdisciplinary simulation system adheres to the concept of functional modular design and employs a model stratification method to achieve the division of data, analysis, and presentation models. This creates a closed-loop practice that spans data sample selection and processing to front-end interaction. Finally, we used a modified version of the System Usability Scale (SUS) questionnaire to evaluate the interdisciplinary simulation system.

**Results:**

Five cases of gout, gastritis, cirrhosis, inflammatory bowel disease, and chronic obstructive pulmonary disease were utilized to master the standard process of data analysis across various datasets from multiple dimensions of the model algorithm, data analysis, and result display.

**Conclusion:**

The “Data-case-simulation” trinity practice teaching model enables students to utilize open-source datasets for case analysis, employing clinical index modeling and statistical thinking. This verifies the efficiency of case simulation analysis within interdisciplinary scenarios and provides a data-driven practice paradigm for medical education innovation. This model holds significant reference value for promoting in-depth cross-disciplinary integration of “medicine + X”.

**Supplementary Information:**

The online version contains supplementary material available at 10.1186/s12909-025-07631-8.

## Introduction

Cultivating interdisciplinary medical talents is now a key goal of medical education. Driven by AI and big data, medical education is shifting from a single - specialty model to one that integrates cross - disciplinary knowledge. This shift helps students break through disciplinary barriers and use interdisciplinary tools to solve real - world clinical problems.

In current medical education, courses usually start with theoretical teaching. In some regions, students first observe or visit hospitals. They then move to medical simulation practice using tools like human models, simulated humans, and standardized patients, and finally transition to virtual simulation [1]. This study positions virtual simulation as an advanced training stage, complementing other training methods.

This research is grounded in China’s medical practice education and closely aligns with the national medical school curriculum, being a crucial part of the teaching reform in internal medicine education.

However, existing medical practice education applications have limitations. For example, disease - centered virtual simulation training [2–4], clinical case practice teaching with artificial intelligence [5, 6], and medical experience courses using VR and serious games [7, 8] mainly use technological means to enrich teaching and enhance visualization. They lack a modular, multi - dimensional analysis approach centered on diseases, making it hard to integrate interdisciplinary applications into the classroom.

To address this, this study introduces the concept of public datasets. It uses a disease - centered approach and a “Medicine + X” interdisciplinary model to build a case - based simulation and analysis system. Based on situated learning theory [9, 10], this system emphasizes practice in real - world and social contexts. It strongly supports our goal of training data - literate clinicians by providing a solid theoretical foundation for the simulation model and learning outcomes, better equipping students for modern evidence - based medicine.

In implementation, this study integrates several authoritative databases, including TCGA [11], GEO [12], and UK Biobank [13]. Using these data resources, students can perform comprehensive data analysis practices, such as differential and survival analysis. In case selection, the study combines disease classifications from hospital departments and uses a component - based approach to build data analysis models, enabling students to master the analysis process across different datasets.

The methodology of this study is significant. It supports students in conducting experiential analysis and carrying out end - to - end data investigations from preprocessing to statistical modeling. This process mirrors diagnostic reasoning in clinical practice and enables a multi - dimensional understanding of diseases through bioinformatics patterns and epidemiological trends. Amid rapid medical development, data literacy is essential for future clinicians [14]. By training students to handle and interpret complex biomedical data, this study enables them to critically evaluate data - driven evidence and integrate multimodal insights in clinical decision - making.

In summary, this study builds on existing medical education practices. By creating an interdisciplinary simulation - based practice system, it offers new approaches for training well - rounded medical talents to meet modern medical needs. Unlike some current changes in medical education [15, 16], this study focuses on future medical education needs, emphasizing data literacy and interdisciplinary collaboration skills. This forward - looking perspective promises to drive the continuous development and innovation of medical education.

## Method

### Data source

The study retrieved data from the following three major databases in February 2024: TCGA, GEO, and UK Biobank. The screening data are detailed in Table 1. TCGA was selected to extract data from six types of tumor samples from 2006 to 2024. The selection criteria of GEO for typical disease samples involve the species being human, and the data type should be a matrix. Clinical indicators of diseases from UK Biobank were selected as data items for modeling analysis. The processing methods for different data sources include:①The simulation analysis method for TCGA and GEO generally adopts the R language, and the advantage is that the simulation analysis strategy [17] can be dynamically adjusted according to the requirements. ②Machine learning modeling for the UK Biobank data uses Python for modeling analysis. The advantage is that Python can provide a rich standard library for users to use [18] and can be quickly transplanted into a interdisciplinary simulation practice system in the form of toolbox packaging. ③A interdisciplinary simulation practice system is constructed using the model stratification method. The advantage is the weak correlation between modules, so that each module can be used independently, allowing students to master the knowledge points [19] at different stages.

**Table 1:** Data items of the case-based interdisciplinary simulation system

Clinical departments	Typical disease	Cause cancer	GSE sample	TCGA sample	Clinical indicators of disease
Endocrinology	Gout	Cholangiocarcinoma	GSE160170	CHOL	Uric acid
Gastroenterology	Liver cirrhosis	Liver cancer	GSE41919	LIHC	Liver stiffness measurement(LSM)
	Gastritis	Gastric cancer	GSE106656	STAD	Helicobacter pylori
	Inflammatory bowel disease	Colorectal cancer	GSE179285	COAD	Fecal calprotectin、occult blood
Respiratory	Chronic obstructive pulmonary disease	Lung cancer	GSE112165	LUAD	Forced Expiratory Volume in the first second(FEV₁)/Forced Vital Capacity (FVC) ratio

### Case selection method in the simulation and analysis system

In the clinical setting of medical students, case-based learning (CBL) is an effective strategy for practical teaching [20]. Proper case selection can significantly enhance the authenticity of the teaching process and ensure that all students can actively engage with the educational content [21, 22]. Consequently, the selection of cases in the construction of a interdisciplinary simulation practice system is particularly crucial, as it involves multiple datasets, each utilizing a distinct analysis algorithm to prevent fragmented analysis of the datasets. Thus, the present study opted for a disease-centered approach, selecting gout, gastritis, liver cirrhosis, inflammatory bowel disease, and chronic obstructive pulmonary disease as teaching practice cases. The aims to illustrate that in the absence of clinical intervention, the disease may progress to malignancy. According to literature review, the aforementioned conditions may develop into cholangiocarcinoma, gastric cancer, hepatocellular carcinoma, colorectal cancer, and lung cancer, respectively [23–27]. Through disease series analysis of different datasets, for instance, in the case of gout, during the differential analysis and enrichment analysis stages, based on gout disease, gene expression data (GSE160170) is determined. In the machine learning modeling stage, using 37 clinical indicators such as uric acid from UK Biobank, a machine learning model is established. During the survival analysis stage, by reporting the understanding that gout may develop into biliary cancer [26], the biliary cancer data from TCGA, CHOL is extracted for analysis. In this manner, through the selection of disease cases, students can master the basic operations of datasets on one hand, and on the other hand, they can learn the standard process of disease-based simulation analysis, encompassing model algorithms, data analysis, and result presentation from multiple dimensions.

### Environmental resources of a interdisciplinary simulation practice system

The hardware environment is supported by the supercomputer public cloud platform of Huazhong University of Science and Technology. Access to the virtual cloud computing server allows for dynamic adjustment of hardware resources based on the data analysis load, facilitating capacity expansion. This provides the necessary hardware support for data analysis from TCGA, GEO, and UK Biobank. The software environment leverages the legalization service platform of Huazhong University of Science and Technology, utilizing the Python server version to simulate UK Biobank. Currently, there are nine virtualized cloud computing servers equipped with two Intel Zhiqiang 20-core CPUs, 512GB of memory, and 21.2 TB SAS hard disks.

### Simulation analysis process based on datasets

Currently, the analysis process within the interdisciplinary simulation practice system is divided into three stages (Table 2). Stage I involves differential and enrichment analyses using the GEO datasets. Stage II conducts survival analysis utilizing the TCGA datasets. Stage III entails constructing and analyzing machine learning models for the diseases identified in the previous two stages. The analysis process is outlined as follows:

**Table 2:** Case-based interdisciplinary simulation system module

Stage	Process steps	Module name
Stage I	(1)Sample matrix data prepared for analysis (the data should include grouping information);	Differential expression genes analysis
	(2)Establish an analysis model based on the grouping information;	
	(3)Select the appropriate algorithm to fit and analyze gene expression levels;	
	(4)The analysis results were obtained, and the topTable was utilized to identify the most significant genes;	
	(5)Based on the differential gene matrix acquired through differential analysis, dynamically adjust the *P*-value and LogFC value to determine the parameter range for the up-regulation and downregulation of differential genes, and annotate each differential gene with its respective up-regulation or downregulation attributes;	
Stage I	(1)The expression matrix of differential analysis is categorized based on three attributes: up-regulated genes, downregulated genes, and stable genes;	Enrichment analysis
	(2)Select the enrichment algorithm to conduct the enrichment analysis on the classified data according to the attributes;	
	(3)Dynamic adjustment of enrichment analysis parameters primarily involves setting the *P*-value for enrichment;	
	(4)Show the enrichment results in a chart form;	
Stage II	(1)Prepare the analytical samples. Obtain matrix data from GEO; clinical patient information and sample survival status data from TCGA;	Survival analysis
	(2)Build a model based on the grouping information;	
	(3)Selection algorithm for differential analysis of gene expression level and clinical data;	
	(4)Based on the results of differential analysis, select the appropriate survival algorithm by establishing a survival model using survival data;	
	(5)Present the results of the difference analysis and survival analysis in chart form;	
Stage III	(1)For the diseases analyzed in the first and second stages, download the clinical biochemical index dataset with the disease at the center;	Machine learning modeling
	(2)Data cleaning, remove the null value data;	
	(3)Select the Python toolkit for machine learning modeling and compare the performance of different models;	
	(4)Identify the strongest clinical indicators correlation for this disease based on the intersection of various models.	

### Simulation technique based on datasets

The interdisciplinary simulation system follows a modular design philosophy and employs a model stratification approach to realize data, analysis, and presentation models. ①The data model involves a process of sample normalization, grouping, and interpretation of the downloaded data. The downloaded sample dataset consists of four parts: the gene expression matrix, clinical data, biochemical metrics, and survival data. The gene expression matrix is utilized to screen for differential genes for differential and enrichment analyses, while the integration of survival data with clinical data is used for prognosis survival analysis of patients. Additionally, the integration of biochemical indicators with clinical data is employed to construct machine learning models. ②The four modules are set as difference analysis, enrichment analysis, survival analysis, and machine learning modeling. Difference analysis involves identifying differential genes by fitting gene expression levels, including formulas (1) and (2). Standard methods for differential analysis are edgeR, limma, and DESeq2, which are three conventional R packages [28–30], and dynamically adjust the *P*-value and LogFC (Fold Change) values (LogFC represents the ratio of expression levels between two samples or groups. After taking the logarithm with base 2, it becomes log2FC. Generally, the default screening criterion for differentially expressed genes is an absolute value of log2FC greater than 1) to determine the genes that are up or down-regulated. The results of the differential analysis are intersected using the intersect method, allowing students to observe the effects of different analysis methods on the outcomes [31]. Enrichment analysis is based on prior datasets and input differential genes for clustering analysis to obtain clustering results. Enrichment analysis includes Gene Ontology (GO) [32]and Kyoto Encyclopedia of Genes and Genomes (KEGG) analyses [33]. The essence of survival analysis is to employ statistical methods to analyze sample survival or mortality [34]. Survival analysis employs Kaplan-Meier(K-M) curves to display the survival or mortality rates of two or more sample groups, utilizing the log-rank test and Cox regression as two complementary statistical inference methods to calculate sample survival outcomes. It primarily analyzes sample survival from both univariate and multivariate perspectives by evaluating the hazard ratio (HR).Machine learning modeling is the process of using machine learning methods to build a disease outcome prediction model based on biochemical indicators in the dataset. Machine learning modeling algorithms include linear regression, support vector machine, and random forest algorithms for students to utilize [35–37].③The presentation model is the outcome display of various modules within the analytical model, including differential analysis that presents volcano plots and differential gene heatmaps. It also showcases the enrichment analysis results of GO and KEGG through graphical forms.

1 $$ {\rm{k1}}\,{\rm{ = }}\,\left( {{\rm{deg\$ P}}{\rm{.Value}}\,{\rm{ < }}\,{\rm{P}}{\rm{.Value\_t}}} \right)\,{\rm{\& }}\,\left( {{\rm{deg\$ logFC}}\,{\rm{ < }}\,{\rm{ - logFC\_t}}} \right) $$

2 $$ {\rm{k2}}\,{\rm{ = }}\,\left( {{\rm{deg\$ P}}{\rm{.Value}}\,{\rm{ < }}\,{\rm{P}}{\rm{.Value\_t}}} \right)\,{\rm{\& }}\,\left( {{\rm{deg\$ logFC}}\,{\rm{ > }}\,{\rm{logFC\_t}}} \right) $$

### Evaluation method of interdisciplinary simulation practice system

The System Usability Scale (SUS), developed by John Brooke in 1986, has proven to be a reliable tool for analyzing the usability of newly developed systems [38]. It utilizes a 5-point Likert scale with 10 items, with half of them positively worded (odd-numbered items) and the other half negatively worded (even-numbered items). Each item is scored from 1 (“strongly disagree”) to 5 (“strongly agree”). The SUS produces a total score ranging from 0 to 100, where higher scores indicate better usability. The total score is calculated by summing all item scores and multiplying by 2.5. Scores above 70 are considered to meet the “acceptable” threshold [39].

Based on the original SUS question wording, we revised specific items to create a modified SUS questionnaire. This adjustment more effectively captures user feedback on system complexity and aligns with real-world application scenarios. A side-by-side comparison between the original SUS wording and the modified SUS wording is presented in Table 3. The complete English version of the modified SUS questionnaire has been uploaded as a Supplementary File.

To test the usability of the interdisciplinary simulation practice system, we conducted the modified SUS questionnaire interviews with clinical medical students (*N* = 71) at Huazhong University of Science and Technology in 2024. The inclusion criteria of students are: 1.Fourth-year medical student specializing in clinical medicine at Tongji Medical College, Huazhong University of Science and Technology; 2.Undergoing an internship rotation in various internal medicine departments; 3.Willing to collaborate in completing the modified SUS questionnaire and sign the informed consent form.

**Table 3:** Side-by-side comparison of the original SUS question wording and the modified SUS wording

Question number	The original SUS question wording	The modified SUS question wording
1	I think that I would like to use this system frequently.	I think that I would like to use this system frequently.
2	I think that I would like to use this system frequently.	I think that I would like to use this system frequently, nor does the integration of multiple datasets appear necessary.
3	I thought the system was easy to use.	I thought the system was easy to use and could potentially contribute to the development of interdisciplinary practice frameworks.
4	I think that I would need the support of a technical person to be able to use this system.	I think that I would need the support of a technical person to be able to use this system.
5	I found the various functions in this system were well integrated.	I found the various functions in this system were well integrated.
6	I thought there was too much inconsistency in this system.	I thought there was too much inconsistency in this system, and it did not align well with theoretical foundations.
7	I would imagine that most people would learn to use this system very quickly.	I would imagine that most people would learn to use this system very quickly.
8	I found the system very cumbersome to use.	I found the system very cumbersome to use.
9	I felt very confident using the system.	I felt very confident using the system.
10	I needed to learn a lot of things before I could get going with this system.	I needed to learn a lot of things before I could get going with this system.

## Results

### Case-based interdisciplinary simulation practice architecture based on datasets

**Fig. 1:**
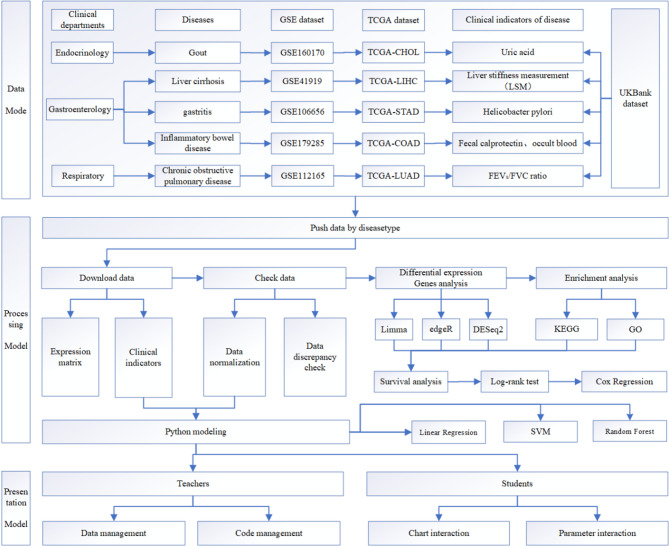
Case-based interdisciplinary simulation practice system diagram

The case-based interdisciplinary simulation practice system, which is data-driven, is composed of data, processing, and presentation models. The entire simulation practice system is depicted in Fig. 1. The data model is constructed using clinical department disease summaries from TCGA, GEO, and UK Biobank. The processing model integrates enrichment analysis, differential analysis, survival analysis, and the standard bioinformatics simulation process using Python modeling. The rendering model is bifurcated into teacher and student components, primarily responsible for dataset management and result display. It dynamically displays differential gene heat maps and presents GO and KEGG enrichment results, as well as survival analysis curves, through graphical means.

### Difference analysis and enrichment analysis using gout as a case study

**Fig. 2:**
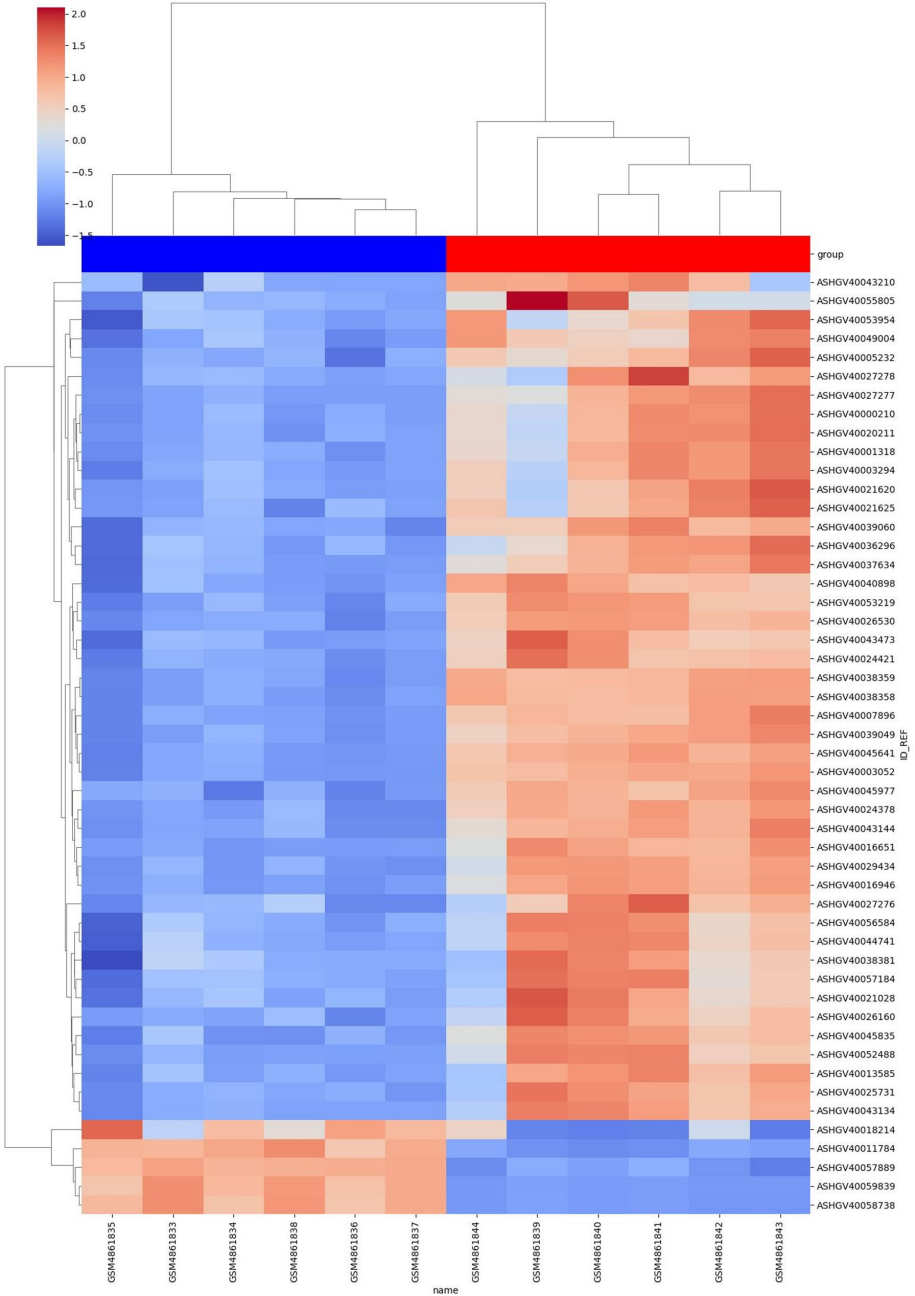
Heat map of the differentially expressed genes analysis

The interdisciplinary simulation practice system offers disease classification patterns. Select the gout data sample GSE160170 to extract the dataset expression matrix. Analysis is conducted using the differential analysis packages of DESeq2, edgeR, and limma. As shown in formulas (1) and (2), where ‘deg’ represents the expression matrix of the differential genes, ‘logFC’ represents the fold change of the difference, and ‘P.Value’ indicates the probability that the screened genes are differentially expressed. Generally, according to statistical definitions, a P.Value of 0.01 indicates a significant statistical difference. When ‘logFC_t’ is set to 1, and ‘P.Value_t’ is set to 0.01, the heatmap of the differential genes is obtained (Fig. 2).

GO enrichment analysis primarily employs the enrich GO function, while KEGG enrichment analysis utilizes the enrich KEGG function by dynamically adjusting the *p*-value to determine the enrichment of biological processes(BP), cellular components(CC), or molecular functions(MF). The GSE160170 sample enrichment results encompass categories of biological processes, pathway enrichment, and subsequent analysis of the target genes related to the clinical effects of gout disease.

### Survival analysis of gout progression to cholangiocarcinoma examples

**Fig. 3:**
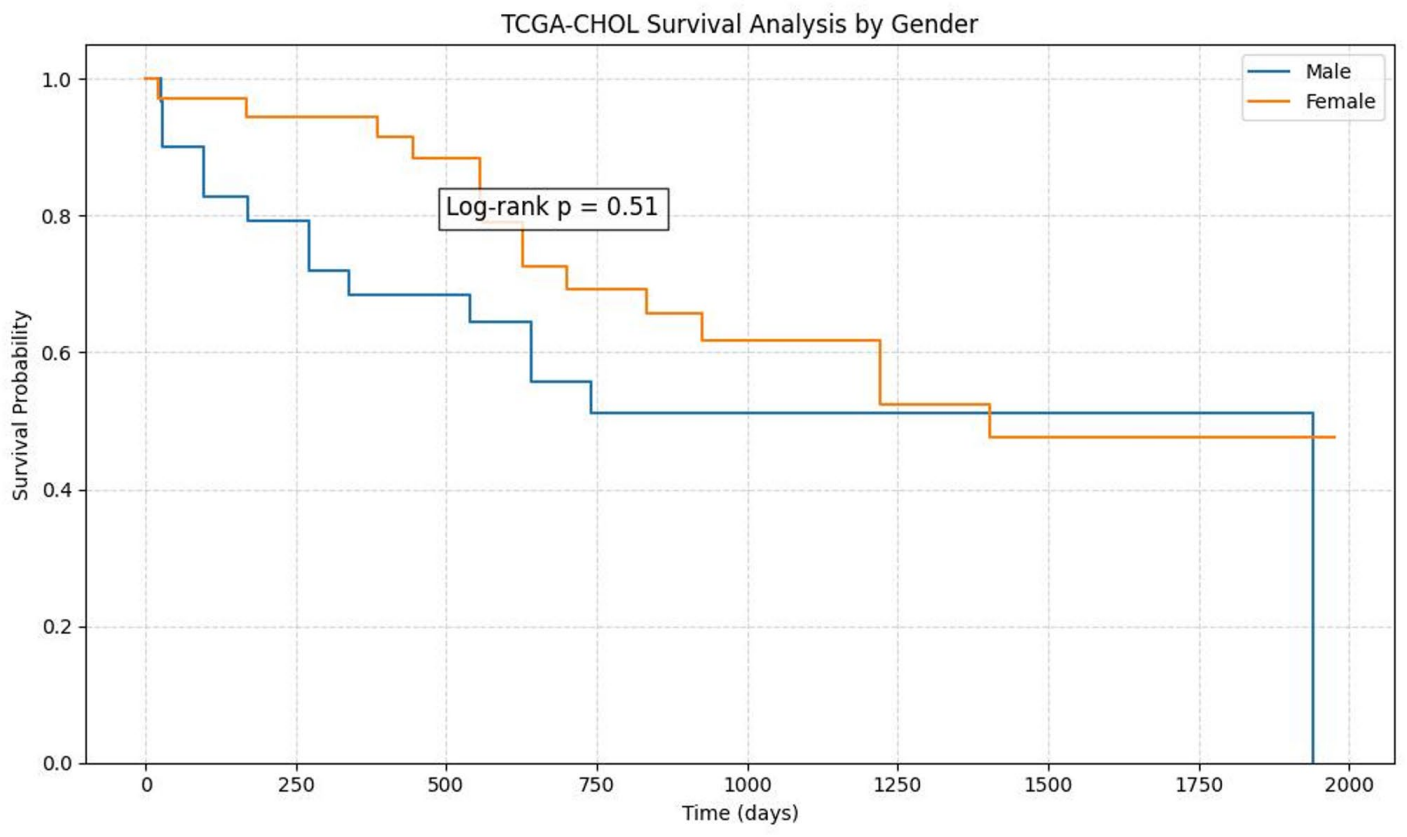
KM survival curves

The interdisciplinary simulation practice system offers a cancer classification mode, utilizing TCGA-CHOL bile cancer data samples. It extracts gene expression matrices, clinical patient data, and sample survival statuses. For survival analysis, the log-rank test and Cox regression are employed. The survfit function, based on the log-rank test, generates survival time and sample survival KM curves. General grouping can be performed by sex, age, and gene, with grouping information requiring discrete values. Students can compare the survival of samples using different *P*-value sizes. For instance, grouping by sex yields a *P*-value effect of 0.51 (Fig. 3).

### Machine learning model using high uric acid as a clinical diagnostic indicator for gout

Utilizing the UK Biobank, 1088 population samples were included, and 37 indicators were established, encompassing biological characteristics and signs, other diseases, physical activity, and clinical laboratory results. The specific indicators are detailed in Table 4.

**Table 4:** Model indicators for machine learning

Index classification	Index item	Index number
Biological characteristics and signs	Gender, Age, Height, Weight, Body Mass Index (BMI, kg/m^2^),Diastolic blood pressure (mmHg), Systolic blood pressure (mmHg)	7
Other diseases	History of diabetes, History of thyroid, Osteoporosis	3
Physical activity	Total weekly activity (hours/week)	1
Clinical laboratory results	Leocytes (10^9 cells/L), Lymphocytes (10^9 cells/L), Monocytes (10^9 cells/L), Neutrophils (10^9 cells/L), AST (U/L), Urea (mmol/L), Blood calcium (mmol/L), Total cholesterol (mmol/L), Creatinine (µmol/L), C-reactive protein (CRP, mg/L), ALT (U/L), Blood glucose (mmol/L), Glycated hemoglobin (mmol/mol), HDL-C (mmol/L), LDL-C (mmol/L), Blood phosphorus (mmol/L), Testosterone (nmol/L), Triglycerides (mmol/L), Uric acid(µmol/L), Vitamin D (nmol/L), Glomerular filtration rate(mL/min/1.73m^2^), LSM(kPa), Helicobacter pylori(+/-), Fecal calprotectin(ug/g), Occult blood(+/-)and FEV1/FVC ratio(%)	26

**Fig. 4:**
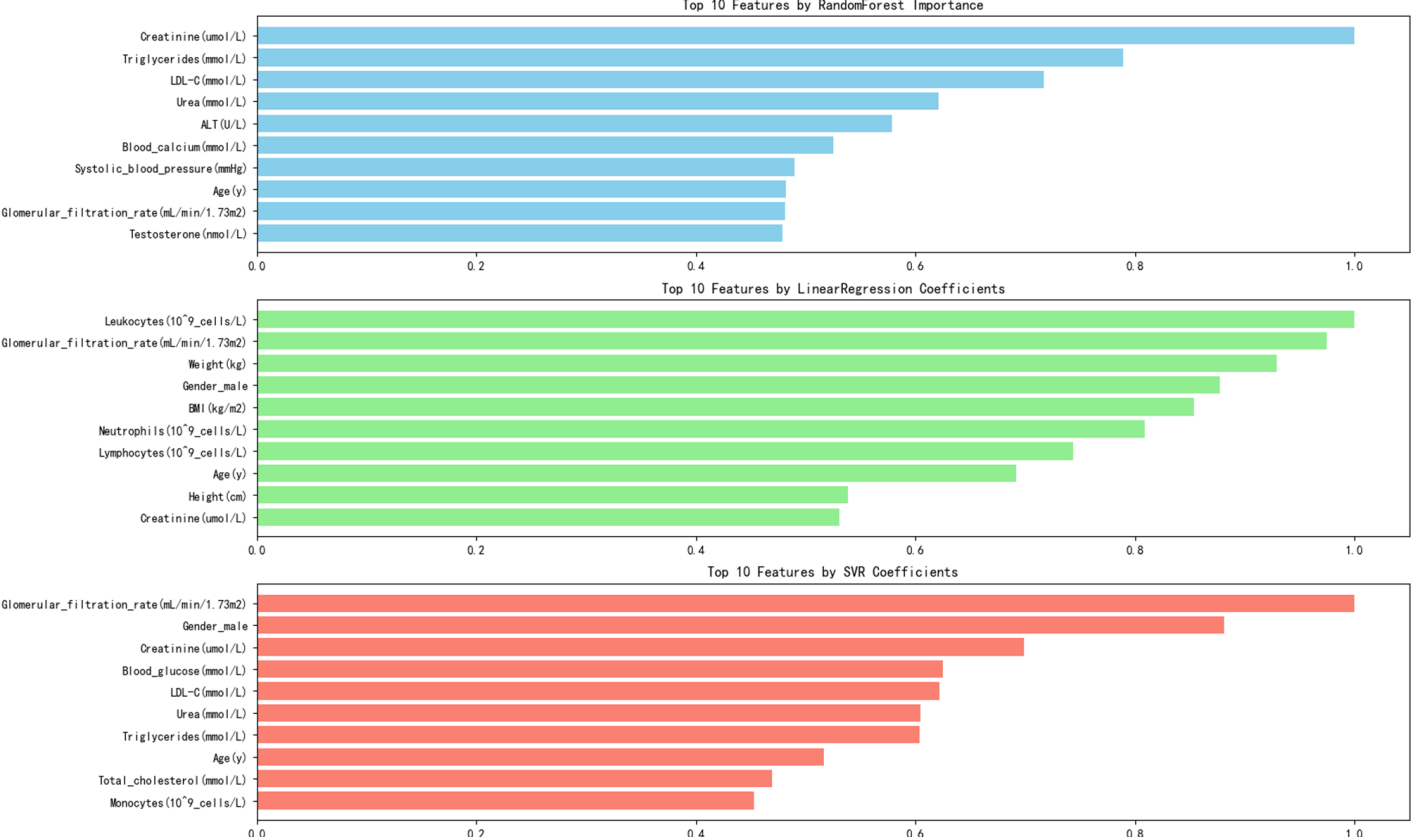
Association analysis of uric acid and clinical indicators

The interdisciplinary simulation practice system integrates the multiple modeling algorithms of random forest, linear regression, and support vector machines. Taking the random forest algorithm modeling as an example, there was a strong correlation between uric acid and creatinine (µmol/L), AST (U/L), and triglyceride (mmol/L). A comparison of the three modeling algorithms using the different algorithms is shown in Fig. 4.

### Evaluation of the interdisciplinary simulation practice system based on the modified SUS

Upon analyzing the modified SUS, the overall performance of the interdisciplinary simulation practice system was found to be excellent, with an average score of 80.77 (out of 100), significantly surpassing the SUS standard average of 68 points and achieving an “excellent” rating (80+). This suggests that students have a very high level of acceptance and satisfaction with the system. Additionally, the system demonstrated good consistency, with a standard deviation of 7.34 points, indicating a high concentration of participant scores. The median score of 80 aligns closely with the average (80.77), and even the lowest score of 72.5 is above the industry average. In terms of score distribution, approximately 65% of the sample scored within the 75–85 point range, with no scores below 70. The scoring outcomes reached the “best acceptable range” of the the SUS scale, signifying that the simulation practice system excels in usability, ease of learning, and user acceptance, making it suitable for promotion and application in interdisciplinary practice teaching.

## Discussion

By integrating public datasets such as TCGA, GEO, and UK Biobank, this study constructed a disease-centric “medicine + X” simulation practice system, effectively addressing the pain point of insufficient interdisciplinary practice in traditional medical education. Compared to existing virtual simulation platforms [2–4], this simulation practice system leverages open-source data and employs modular design to facilitate the entire process analysis, from data preprocessing to result display.

From a technical standpoint, students employed a standardized simulation analysis process and modular function division to enhance their comprehension of the simulation analysis process for open-source datasets. The simulation practice system in this study is characterized by its diverse analysis models, streamlined analysis process, and visualization of analysis results. Among the various analytical models, students are provided with a selection of embedded multiple analysis algorithms. The experimenter can model diseases and choose to employ different algorithms to compare the analysis effects of diseases under various algorithms (such as DESeq2, edgeR, or the Python machine learning toolbox in R language). The analysis process is modularized, allowing for the flow-through of differential analysis, enrichment analysis, and survival analysis, which are broken down into steps for convenience in strengthening students’ understanding of theoretical knowledge points and deepening their grasp of the practical operation analysis steps. Visualization of the analysis results is also provided, with all intermediate process data and result data available for download and displayed to users in graphical forms (e.g., heat maps, KM survival curves), facilitating further analysis and use by students with other means and technologies. This simulation practice system not only lowers the technical threshold but also fosters systematic thinking in students through a standardized process that spans from differential gene screening, enrichment analysis, survival association, to model construction, thereby circumventing the drawbacks of traditional virtual simulation platforms that “emphasize operation and neglect logic” [40, 41].

Regarding dataset selection, current medical public open-source large datasets are characterized by reliable data sources, high data quality, and large sample sizes, encompassing various disciplines such as clinical medicine, public health, and biological information. In the realm of public health, exemplified by UK Biobank, these datasets include extensive basic structured data, high-throughput genomics genetic data, and multimodal imaging data. Many scholars depend on the research and analysis [13] of the environment and diseases. In the field of clinical medicine, represented by TCGA, there is a quantitative and qualitative analysis of diseases [42]. GEO is the most widely used database in bioinformatics, covering gene expression matrix data [43]. It is evident that data are ubiquitous in the domain of medical applications, with vast amounts of data being utilized to identify key disease targets or to integrate data for cross-sectional analysis. Fundamentally, the aforementioned analysis fosters students’ analytical skills through a practical learning approach. This practical learning model aligns highly with [20] the CBL method proposed by Cen et al., which confirms that active exploration based on real data enhances students’ ability to solve complex clinical problems. In this study, students were required to integrate multiple datasets in actual case analyses, initially screening for related differential genes from GEO, and subsequently using UK Biobank to construct a disease-centric prediction model. UK Biobank incorporated uric acid, creatinine and other 35 clinical indicators, while TCGA was used to verify the association between disease and cancer survival rates as reported in the literature. This progressive design enables students to fully comprehend the disease, from molecular mechanisms to clinical prognosis, bridging the gap between bioinformatics and clinical medicine in traditional teaching.

When utilizing feedback, the university public information service platform employs hardware to provide server and force support for data analysis. Compared to traditional virtual simulation platforms, such as the oral pulpotomy simulator [2] or the VR technology used to establish a virtual clinic [7], our system relies on high-precision modeling and special equipment. However, a virtual simulation system based on open-source data and general analysis tools (like R/Python) significantly reduces hardware costs. The modified SUS evaluation results indicate that this virtual simulation system’s usability score is 80.77 points, surpassing the industry standard of 68 points. There are no low segment samples, suggesting that its interface friendliness and functional integration are widely recognized by students. Practice has shown that this simulation practice system places greater emphasis on data-driven logic training and data operation simulation, aligning with the demands of modern medical research for compound talents. Looking ahead, we can further integrate generative AI models and provide real-time data analysis code and case generation functions. By developing a graphical interactive interface, we aim to reduce the learning curve for non-information students.

While our platform is designed as a data-driven analytical tool rather than a conventional clinical simulator (e.g., manikin-based scenarios or standardized patient interactions), it actively complements clinical simulation paradigms by cultivating critical competencies in medical data science. Specifically, the system: Anchors learning in clinical contexts: Students engage with real-world, high-incidence diseases through rigorously curated public datasets. Supports experiential analysis: Learners conduct end-to-end data investigations—from preprocessing to statistical modeling—mirroring the diagnostic reasoning process in clinical practice. Enriches theoretical knowledge: Results generated serve as evidence-based extensions to textbook content, enabling students to understand diseases multidimensionally (e.g., via bioinformatic patterns, epidemiological trends). We emphasize that this system is deployed alongside—not as a replacement for—traditional simulation methods. Our approach responds to medicine’s evolving demands: data literacy is now indispensable for future clinicians. By training students to interpret complex biomedical data, we prepare them to critically appraise AI/omics-driven evidence, and synthesize multimodal insights during clinical decision-making.

### Limitations

While the system excels in interdisciplinary integration, it does have certain limitations. Firstly, the dataset is restricted and should be expanded to include more common diseases to enhance future teaching scenarios. Secondly, the steep learning curve associated with technical tools, such as the Python language, may deter non-technical students from participating. Furthermore, the evaluation primarily relied on subjective feedback provided by students via the modified SUS, without incorporating objective metrics to directly assess teaching effectiveness. However, these objective metrics hold significant value for validating the system’s educational impact, so this omission indeed constrains our study. Lastly, the scope of the dataset, sample size, or potential bias in participant recruitment (e.g., selecting for data - analysis affinity, which may distort SUS scores). While the system is a valuable tool in the “data-case-simulation” teaching framework and helps students integrate interdisciplinary tools and enhances their understanding of clinical prognosis, it’s just one component in the broader landscape of interdisciplinary talent development.

## Conclusion

This study integrates modular, layered design (encompassing data, analysis, and presentation models) with standardized analysis workflows (including differential, enrichment, and survival analyses, as well as machine learning modeling). Through five disease case studies (such as gout and gastritis), students can master the integration of multi-disciplinary tools, thereby validating the feasibility of the “data-case-simulation” teaching model.

Innovatively, the study employs open-source medical big data to connect multi-dimensional dataset analyses across various diseases. For example, in the gout case, it links GSE160170 for differential gene screening, UKB for biochemical modeling, and TCGA-CHOL for survival validation, thus bridging bioinformatics and clinical medicine.

The modified SUS evaluation suggests the system is usable, supporting its potential for further development in medical education. It enables students to rapidly adapt to interdisciplinary scenarios and apply tools such as R and Python to real-world medical problems. Consequently, it offers a reproducible teaching framework for cultivating versatile medical professionals. It also provides a data-driven paradigm for medical education innovation and holds significant reference value for promoting the deep integration of “Medicine + X”.

## Electronic supplementary material

Below is the link to the electronic supplementary material.

Supplementary Material 1

## Data Availability

Sequence data that support the findings of this study have been obtained from TCGA and GEO with the datasets GSE160170、GSE41919、GSE106656、GSE179285、GSE112165，CHOL、LIHC、STAD、COAD、LUAD. The UK Biobank data requires a paid download to obtain. The datasets used and/or analysed during the current study available from the corresponding author on reasonable request.

## Abbreviations

BMI: Body Mass Index
BP: Biological processes
CB: Case-based learningt
CC: Cellular components
CRP: C-reactive protein
FEV₁: Forced Expiratory Volume in the first secondt
FVC: Forced Vital Capacityt
GEO: Gene Expression Omnibus
GO: Gene Ontologyt
HR: Hazard ratio
K-M: Kaplan-Meiert
KEGG: Kyoto Encyclopedia of Genes and Genomest
LSM: Liver stiffness measurement
MF: Molecular functions
NCI: National Cancer Institute
NHGRI: National Human Genome Research Institute
SUS: System Usability Scale
TCGA: The Cancer Genome Atlas
