# Supplementary material for: Interdisciplinary medical education practices: building a case-driven interdisciplinary simulation system based on public datasets
Source: BMC Med Educ. 2025 Jul 11;25:1037. doi: 10.1186/s12909-025-07631-8 (PMC12255017; doi:10.1186/s12909-025-07631-8)
Supplement: Supplementary file 1 — Supplementary Material 1 [file 12909_2025_7631_MOESM1_ESM.pdf]

The modified System Usability Scale (SUS) questionnaire for the interdisciplinary simulation practice system

一、I think that I would like to use this system frequently.

☐ 1（Strongly disagree）☐2（Relatively disagree） ☐3（Neutral）
☐4（Relatively agree） ☐5（Strongly agree）

二、I think that I would like to use this system frequently, nor does the integration of multiple datasets appear necessary.

☐ 1（Strongly disagree）☐2（Relatively disagree） ☐3（Neutral）
☐4（Relatively agree） ☐5（Strongly agree）

三、I thought the system was easy to use and could potentially contribute to the development of interdisciplinary practice frameworks.

☐ 1（Strongly disagree）☐2（Relatively disagree） ☐3（Neutral）
☐4（Relatively agree） ☐5（Strongly agree）

四、I think that I would need the support of a technical person to be able to use this system.

☐ 1（Strongly disagree）☐2（Relatively disagree） ☐3（Neutral）
☐4（Relatively agree） ☐5（Strongly agree）

五、I found the various functions in this system were well integrated.

☐ 1（Strongly disagree）☐2（Relatively disagree） ☐3（Neutral）
☐4（Relatively agree） ☐5（Strongly agree）

六、I thought there was too much inconsistency in this system, and it did not align well with theoretical foundations.

☐ 1（Strongly disagree）☐2（Relatively disagree） ☐3（Neutral）
☐4（Relatively agree） ☐5（Strongly agree）

七、I would imagine that most people would learn to use this system very quickly.

☐ 1（Strongly disagree）☐2（Relatively disagree） ☐3（Neutral）
☐4（Relatively agree） ☐5（Strongly agree）

八、I found the system very cumbersome to use.

☐ 1（Strongly disagree）☐2（Relatively disagree） ☐3（Neutral）
☐4（Relatively agree） ☐5（Strongly agree）

九、I felt very confident using the system.

☐ 1（Strongly disagree）☐2（Relatively disagree） ☐3（Neutral）
☐4（Relatively agree） ☐5（Strongly agree）

十、I needed to learn a lot of things before I could get going with this system.

☐ 1（Strongly disagree）☐2（Relatively disagree） ☐3（Neutral）
☐4（Relatively agree） ☐5（Strongly agree）
